# Displaced risk. Keeping mothers and babies safe: a UK ambulance service lens

**DOI:** 10.29045/14784726.2023.9.8.2.52

**Published:** 2023-09-01

**Authors:** Stephanie Heys, Camella Main, Aimee Humphreys, Rachael Torrance

**Affiliations:** North West Ambulance Service NHS Trust; University of Central Lancashire https://orcid.org/0000-0002-4379-9022; London Ambulance Service NHS Trust; London Ambulance Service NHS Trust; North West Ambulance Service NHS Trust

**Keywords:** governance, maternity, neonate, paramedic, pregnancy, pre-hospital, safety

## Abstract

**Aim:**

The aim of this professional practice paper is to provide a critical commentary on displaced risk among perinatal and neonatal patients attended to by the ambulance service.

**Background:**

NHS services across the United Kingdom are currently facing unprecedented demand and increased scrutiny in their ability to provide safe and personalised care to patients. While current focus in the system centres around addressing social care demand, hospital bed capacity, planned care waiting times, staffing and ambulance handover delays, a less explored cohort of patients impacted by the current healthcare crisis is perinatal and neonatal populations attended to by the ambulance service. Little focus has been paid within national agendas to the care provided to women and babies outside of planned maternity and obstetric care. A case is presented to highlight the importance of considering urgent and emergency maternity care provision provided by the ambulance service, and the impact of ‘displaced risk’ due to the current pressures within healthcare systems.

**Conclusion:**

Placed in a national context, drawing upon current independent reviews into maternity services, national transformation agendas and the most recent MBRRACE-UK confidential enquiry into maternal deaths and morbidity, a case is made to commissioners and Integrated Care Systems to focus on and invest in the unplanned pre-hospital care of maternity and neonatal patients. Recognition of the ambulance service as a key provider of care to this cohort of patients is paramount, calling on services and systems to work together on realising and addressing displaced risk for perinatal populations across the United Kingdom. A system approach that acknowledges the need for high-quality care at every point of contact and equitability in access to services for pregnant, postpartum and neonatal patients is vital.

## Safety in UK maternity care: current picture

Maternity services across the United Kingdom are currently facing unprecedented demand, staffing pressures and increased scrutiny in their ability to provide safe and personalised care to women and babies. Several historical and ongoing independent investigations into NHS maternity services have been published (Dixon-Woods, 2022; Kirkup 2015, 2022), highlighting ongoing concerns for patient safety. In 2022, Donna Ockenden released the final report following maternity care failings at Shrewsbury and Telford Hospital NHS Trust. The report provided local actions for learning and immediate and essential actions (IEAs) to improve safety across all maternity services in England (Independent Maternity Review, 2022). However, the report is clear that all NHS trusts should assess their service against IEAs, recognising that similar failings and patient safety concerns are likely evident on a national scale, not just within maternity services. The East Kent report further highlighted key themes that compromised maternal and neonatal safety (Kirkup, 2022). Both reports echo similarities with historical clinical and cultural failings at Morecambe Bay, highlighting concerns regarding the responsibility and accountability of NHS trusts in addressing systemic failings within maternity care (Knight & Bevan, 2021; Knight & Stanford, 2022). Central to this commentary is the notion of displaced risk, referring to the impact of pressures within healthcare systems that may result in women accessing services outside of current provisions of maternity care.

In 2021, the *BMJ* published a seminal piece detailing the seven features of safety in maternity units, outlining behaviours and practices identified as key features of safe care in hospital-based maternity units (Liberati et al., 2021). Aligned to the recent Health and Social Care Committee (2021) publication, *The safety of maternity services in England*, recommendations across both publications are clear: cross-collaborative, multidisciplinary and woman-centred personalised care reduces maternal and neonatal mortality and morbidity. Following a detailed and wide-ranging review of maternity services by the Health and Social Care Committee, the report failed to address ambulance service involvement in the care women and babies receive outside of definitive places of care. According to the Birthplace in England Collaborative Group (2011), for women having a first baby who have chosen to birth at home with care provided by a midwife, the likelihood of transferring via ambulance from home to an obstetric unit during labour or immediately after the birth is 45%. For women having a second, third or fourth baby, the rate is around 10%. Coupled with women who access emergency and urgent care in the absence of midwifery support, a focus on safe, personalised clinical care provided to perinatal populations via the ambulance service is well overdue.

Pre-hospital maternity care is often aligned to that of women making an informed choice to birth in a low-risk environment, such as a midwifery-led unit or in the home, supported by midwives. A less explored cohort of women is those attended to by the ambulance service, in the absence of a midwife following an unplanned event during labour and birth, or those requiring an emergency transfer into an obstetric unit from a free-standing unit or homebirth setting. Furthermore, not all maternity calls relate to birth; some of the most complex cases attended to by the ambulance service relate to emergencies throughout the childbearing continuum and following birth (McLelland et al., 2018).

While the number of babies born in the presence of the ambulance service is not defined across the United Kingdom, a recent study via the South Western Ambulance Service NHS Foundation Trust highlighted that the service attended to 1.5 births a day on average (Goodwin et al., 2022). While data on unplanned births attended to by the ambulance service are helpful, the North West Ambulance Service alone received over 10,000 maternity-related 999 calls during 2022, with the London Ambulance Service receiving around 13,000 maternity-related calls via 999 in the same period, highlighting the variety of presentations attended to among this cohort of patients. While most planned births in the pre-hospital setting are uneventful (Birthplace in England Collaborative Group, 2011), births that require emergency assistance via the ambulance service are associated with adverse outcomes for women and babies (Loughney et al., 2006; McLelland et al., 2018; Thornton & Dahlen, 2018; Unterscheider et al., 2011). Moreover, managing compromised neonates at a range of gestations in an uncontrolled environment is associated with an increase in mortality and morbidity (Boland et al., 2018; NHS England & NHS Improvement, 2019). Despite ambulance services being required to provide urgent and emergency maternity and neonatal care, little focus is paid within national maternity reports regarding care provided to this cohort of patients by the emergency services (NHS England, 2023).

The overall frequency of maternity incidents in comparison to non-maternity calls often results in skill decline and low clinical confidence in managing emergency maternity and neonatal presentation (Heys et al., 2022). Coupled with identified gaps relating to a lack of training and exposure and suitable equipment to enable optimum care provisions for babies born prematurely (Goodwin et al., 2022), findings encourage us to ask how national agendas consider the unplanned pre-hospital setting as a focused area to reduce mortality and morbidity among this cohort of patients. While the recent COVID-19 pandemic has highlighted the need for a re-envisaged NHS (Anderson et al., 2021), the identification of increased healthcare demand over time (Appleby, 2019) and a worrying trend in unsafe maternity staffing levels (NHS England, 2021a), more focus is needed on how system pressures affect perinatal populations and the potential for displaced risk within the pre-hospital setting.

## Equality and equity of care provision for women who access emergency services

Supporting women within the pre-hospital setting who may face disadvantages in terms of unexpected deterioration, barriers and difficulties in engaging with maternity services and those experiencing abject poverty should serve as a starting point for assessing risk, safety and service delivery need among this patient group. The national equality and equity strategy is clear: local maternity and neonatal systems must take decisive action on a range of areas to ensure that equity of outcome is achieved and preventable mortality is reduced among maternity and newborn patients (NHS England, 2021b).

Disparities in maternity outcomes in the United Kingdom have been well-evidenced in recent years. Women facing severe and multiple disadvantages are less likely to attend routine care (Farrant et al., 2022) and are at an increased likelihood of seeking urgent or emergency medical assistance during and following pregnancy (Knight et al., 2022). The most recent confidential enquiry into maternal deaths (Knight et al., 2022) highlighted that inequalities persist for Black and Asian women, with the pandemic further highlighting inequity in maternity services for Black and ethnic minority women and those living in areas of deprivation (Birthrights, 2022). Acknowledging the existing inequalities within maternity care provision, it should also be considered that women who face barriers in accessing maternity services may first present to emergency services (Konje & Konje, 2021). While more research is needed to explore outcomes for women who are attended by the ambulance service, a collaborative focus on co-ordinating care efforts across services is recommended, to ensure every point of care for women and babies during and following pregnancy is acknowledged.

Ambulance services provide care across diverse geographical footprints, serving some of the country’s most deprived communities. Reducing variation in experience or outcome for populations who access healthcare is a focus of most trusts’ quality improvement strategies and national drivers for maternity care delivery (Care Quality Commission, 2022). Despite such a vision, current pressures across the NHS have resulted in a greater equality and equity divide, with risk being displaced into the pre-hospital setting for those under-served by the healthcare systems.

## System-level considerations for safer maternity care: integrated working

A recent Healthcare Safety Investigation Branch (2021) report identified areas for improvement for pre-hospital maternity care, recommending that the Association of Ambulance Chief Executives work together with ambulance services to share best practice to support standardisation and enhance quality care provision.

In 2020, the suspension of homebirth provision across several maternity trusts was publicly documented, and highlighted the importance of system working to address the pressures and impact of withdrawing service provisions (Brigante et al., 2022). A less-documented concern during this time was the expectation of the ambulance service to provide unplanned maternity care to women in their home setting. Given the minimal clinical exposure and low confidence of ambulance clinicians as previously described, such situations often result in extended on-scene times for the ambulance service and increased risk for women due to the lack of attendance by clinicians skilled in providing maternity care. Current demands within healthcare have seen ambulance services absorb a disproportionate level of risk across a number of patient groups (Hopson, 2021), encouraging us to ask commissioned services to acknowledge the need for shared accountability and a collaborative strategy.

As part of the development of Integrated Care Systems, a focus is placed on developing provider collaborations at scale (Department of Health and Social Care, 2022). Historically, ambulance trusts have been excluded from policy making at system level, with the pre-hospital journey not considered outside of the arrival at the emergency department or following ‘see and treat’ assessment in the patient’s home. In the context of maternity and neonatal patients, liaison with healthcare systems via the ambulance service and collaborative working within and across Integrated Care Systems remains disjointed at best. Such findings are not surprising, with a recent regulation 28 from the HM Coroners’ Court (2022) highlighting the stark gap of maternity representation within ambulance services across the United Kingdom as a key safety risk. It is crucial that systems acknowledge that maternity and neonatal patients carry an exponential risk of adverse perinatal outcomes when attended to by the ambulance service (Javaudin et al., 2019), with local maternity and neonatal networks ideally placed to identify variation in outcomes and take action to address local population needs (NHS England & NHS Improvement, 2019). However, variations are noted in the approach taken across systems, the way in which the ambulance service is included in local-level care delivery considerations and conversations around the impact of pressures upon outcomes. Such variation highlights the complexities and difficulties faced by the ambulance service, which often spans several regional footprints, clinical networks and Integrated Care Systems, and presents the reality of ensuring due representation across several systems and services.

## Conclusion

The recognition of the ambulance service as a key care provider of urgent and emergency care to pregnant women and neonates in the pre-hospital setting is paramount. Collaborative endeavours to enhance safety among this cohort of patients are needed to ensure services work together to address some of the United Kingdom’s most pressing healthcare challenges. With inequalities in perinatal outcomes at the forefront of national agendas to improve care for vulnerable groups, such considerations must be placed in the context of emergency and urgent care and how services work across sectors to support individualised care for high-risk women and babies. While a focus on enhancing maternity care provisions is needed, a system approach that acknowledges the need for high-quality care at every point of contact and equitability in access to services across the childbearing continuum is welcomed. Acknowledging that transformational patient safety agendas within maternity care are underway, the pre-hospital setting and emergency service care provision for women and neonates is largely under-explored. This article calls for systems and NHS organisations to ensure that maternity and neonatal care provided by the ambulance service is at the forefront of current and future service redesign to address displaced risk for perinatal populations across the United Kingdom.

## Author contributions

SH acts as the guarantor for this article.

## Conflict of interest

None declared.

## Funding

None.
